# Learning-Based Control of Autonomous Vehicles Using an Adaptive Neuro-Fuzzy Inference System and the Linear Matrix Inequality Approach

**DOI:** 10.3390/s24082551

**Published:** 2024-04-16

**Authors:** Mohammad Sheikhsamad, Vicenç Puig

**Affiliations:** Institute of Robotics and Industrial Informatics (CSIC-UPC), Llorens i Artigas, 4-6, 08028 Barcelona, Spain; mohammad.sheikhsamad@upc.edu

**Keywords:** ANFIS controller, linear matrix inequality, Takagi–Sugeno, autonomous driving

## Abstract

This paper proposes a learning-based control approach for autonomous vehicles. An explicit Takagi–Sugeno (TS) controller is learned using input and output data from a preexisting controller, employing the Adaptive Neuro-Fuzzy Inference System (ANFIS) algorithm. At the same time, the vehicle model is identified in the TS model form for closed-loop stability assessment using Lyapunov theory and LMIs. The proposed approach is applied to learn the control law from an MPC controller, thus avoiding the use of online optimization. This reduces the computational burden of the control loop and facilitates real-time implementation. Finally, the proposed approach is assessed through simulation using a small-scale autonomous racing car.

## 1. Introduction

*Autonomous driving* is one of the top ten technologies that will change the lives of citizens, according to the European Parliament Research Service (EPRS) [1]. The National Highway Traffic Safety Administration (NHTSA) currently states in its technical report that 94% of road accidents are the consequence of human error [2]. By 2050, it is projected that 68% of the world’s population will live in urban regions, up from 55% in 2018 [3], increasing traffic congestion. Autonomous driving emerges as a solution to these challenges, and it facilitates the following [4]:

- Attaining close to zero traffic accidents.
- Improving accessibility for people with low physical mobility.
- Lessening congestion through shared routes for both passengers and goods, coupled with intelligent motion.
- Lowering energy consumption and pollution [1].

Consequently, industrialized countries are actively engaged in a competitive race to develop autonomous driving technology, and leading research institutions and companies are achieving great success. Recent progress in software (artificial intelligence, planning and control, telecommunications, etc.), hardware (sensors, embedded supercomputers, etc.), laws, and user acceptance suggests that autonomous driving is just a matter of time, although achieving full autonomy presents many challenges.

To address these challenges, the Society of Automotive Engineers (SAE) outlines five progressive levels of automation [5], ranging from driver assistance (level 1) to full autonomy (level 5). In levels 2 through 5, the autonomous vehicle driving system is in charge of steering, braking, and accelerating. This autonomous driving system consists of multiple components that necessitate seamless integration to operate as a cohesive unit. These components include perception, motion planning, vehicle localization, pedestrian detection, traffic-sign detection, road-marking detection, automated parking, vehicle cyber security, fault diagnosis, and automatic control. Automatic control is in charge of driving the vehicle between two points as well as generating smooth control actions to ensure a comfortable ride [6] by controlling the lateral and/or longitudinal dynamics. This is a challenging task that puts the vehicle into motion using sensors (GPS, IMU, encoders, cameras, LIDAR, etc.) to measure the environment and the vehicle variables, and then provides the appropriate signals to the actuators (steering motor, electric engine, and braking system).

To address the problem of automatic control in autonomous driving systems, a variety of automatic control strategies have been developed and implemented, e.g., proportional–integral–derivative (PID) control [7,8,9], robust control ($H_{\infty }$) [10], fuzzy logic control [7,11,12], sliding-mode control (SMC) [13,14], Lyapunov-based control [14,15], linear parameter-varying (LPV) control [16], Takagi–Sugeno control [17], and linear quadratic regulator (LQR) control [18]. Model predictive control (MPC) stands out as a highly effective control strategy, relying on the dynamic model of the system to anticipate future states. Thus, MPC has the capability to predict upcoming events and take appropriate control actions. It is based on an optimal control law that minimizes a cost function in real time during each iteration. Numerous notable efforts have been undertaken in this field [19,20,21,22]. Since the vehicle model is non-linear, non-linear MPC (NL-MPC) can be applied but real-time implementation is still an issue because of the small sampling times used [23,24,25,26].

The linear parameter-varying (LPV) approach mentioned in [27] is a control strategy utilized in many control applications (see [16] for a recent review). It enables the transformation of a non-linear system into a linear-like representation by embedding the system’s non-linearities inside variable parameters. LPV-MPC [28,29] uses LPV to obtain the vehicle model, and despite its merits, the control still needs online optimization for MPC during iteration, but with less computational load than NL-MPC. Another versatile and effective tool in automatic control is the Linear Matrix Inequalities (LMIs) approach [30], which allows for systematic design of closed-loop systems with guarantees of stability and performance without requiring online optimization [31,32,33].

The Adaptive Neuro-Fuzzy Inference System (ANFIS) [34] is a learning approach that synergizes the capabilities of two soft computing frameworks: Artificial Neural Networks (ANNs) and fuzzy logic (FL). The ANFIS can model complex, non-linear functions that may not be easily described using physical mathematical equations. This model can also be represented explicitly and interpreted in Takagi–Sugeno (TS) form. Several studies have used ANFISs in various applications. Ref. [35] applied an ANFIS to enhance vehicle route selection in uncertain conditions. Ref. [36] aimed to build an ANFIS driver model that could replace a real one. Ref. [37] used an ANFIS for navigation and target acquisition for an autonomous robot in both static and dynamic environments. Ref. [38] applied an ANFIS to establish a systematic process to access the complex operations of working vehicles. Ref. [39] proposed an ANFIS to design a controller with self-position azimuth correction (SPAC) for trajectory tracking and obstacle avoidance. Ref. [40] presented an ANFIS-based approach to mobile robot navigation and obstacle avoidance in unknown static environments, considering obstacle distances and steering angles as the ANFIS input and output, respectively.

The TS fuzzy modeling approach, as proposed by Takagi and Sugeno [41], serves as a universal approximator for any smooth non-linear system [42]. It provides a systematic approach for generating fuzzy rules from input and output data, and it is able to represent the local dynamics of each fuzzy rule through a linear system model. This enables the representation of a large family of non-linear dynamical systems with a high degree of precision. Using the TS approach, several notable developments are actively moving forward [17,43,44]. One common application is the use of the TS model to represent the *vehicle model* for control tasks [45,46]. Ref. [47] proposed TS MPC for motion planning. However, in both cases, the controller/planner still needs to perform online optimization to minimize the MPC cost function during each iteration using the vehicle TS model. To the best of the authors’ knowledge, there is a lack of evidence supporting the direct application of TS to learn the control law (*control model*) with stability guarantees.

This paper proposes a learning-based controller for autonomous vehicles. We use the ANFIS as a learning method to directly obtain control laws from data (*control model*) in TS model form. Another TS model is obtained to represent vehicle behavior (*vehicle model*). The application of TS representation for the vehicle model has already been considered for control design in the literature. However, this paper uses this model to verify the closed-loop stability of the learned TS controller in an innovative manner using Lyapunov theory and LMIs. A reliable working controller (in this paper, an MPC controller) is used as a data generator to provide the required input/output data for obtaining a TS model for the controller using the ANFIS. The proposed approach is validated through simulation with a small-scale autonomous race car. The obtained results show that the proposed approach has the merit of reducing computational complexity by removing online optimization.

This paper is organized as follows. Section 2 outlines the proposed approach. Section 3 provides details on the learning-based control design and introduces the autonomous vehicle considered as a case study. The simulation results are presented in Section 4. Finally, Section 5 summarizes the key findings of this paper and suggests potential paths for future research.

## 2. Proposed Approach

**Figure 1:**
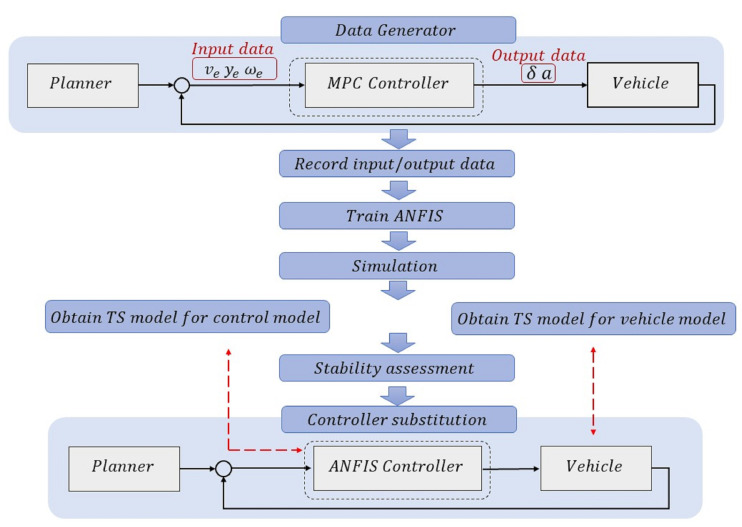
Introduction of the main idea and proposed approach.

The core concept behind the proposed approach is to develop a controller for an autonomous vehicle by relying on machine learning and data rather than conventional model-based control strategies, which mostly rely on physical models. When employing the ANFIS as a machine learning method, the resulting controller is referred to as the ANFIS controller in the remainder of this paper. It is used as a feedback controller to provide appropriate control actions (controller output) to carry out the planned motion and correct tracking errors (controller input). Tracking errors are generated during the execution of a planned motion. Hence, the term “data” refers to the input and output values of the controller, and the objective is to design a controller based only on these data. Various methods exist to generate data, including simulation (using different control strategies) or conducting real-world experiments (application of various control actions to the vehicle). In this work, as shown in Figure 1, an existing MPC controller is used as a data generator. This means that there already exists an autonomous vehicle system that works with an MPC controller (previously designed and validated in [47]). This MPC controller functions effectively, and throughout its operation, the input and output data are recorded. By using these data to train an ANFIS structure, we can obtain an ANFIS controller. In other words, this ANFIS controller is intended as a potential substitute for the MPC controller, and a comparison of the operations and parameters of these two controllers is investigated. Furthermore, the stability of the new autonomous vehicle system using the ANFIS controller is examined. It should be noted that for stability assessment, both the control and vehicle models must be derived from two distinct TS representations.

The proposed approach comprises the following steps:

To design such a machine learning-based control strategy and ensure closed-loop stability, the following procedure is briefly outlined:

- Step 1: Generate the input and output data.
- Step 2: Employ the ANFIS to learn the control law from the data.
- Step 3: Validate the learned controller through simulation.
- Step 4: Obtain the TS model of the control model and vehicle model.
- Step 5: Stability proof of the closed-loop system.

### 2.1. Generate the Input and Output Data

The initial step consists of acquiring the data. Regardless of the data generation method used, the quality of the data directly influences the effectiveness of the controller. In this paper, an MPC controller functions as a data-generating tool. The controller inputs are the tracking errors measured during the operation of the autonomous vehicle using the MPC controller, $x_{c}=\left[v_{e}\;y_{e}\;\theta_{e}\right]$, which are the errors in the longitudinal speed, lateral speed, and angular velocity of the vehicle, respectively. The controller outputs are the control actions of the MPC controller, $u={\left[\delta \;a\right]}^{T}$, which correspond to the steering wheel angle and acceleration applied to the autonomous vehicle, respectively.

### 2.2. Employ ANFIS to Learn the Control Law from the Data

The ANFIS is used to learn a control law from the input and output data. This modeling tool configures a neural network that learns the dynamic behavior of the vehicle using the backpropagation technique and the least squares (RLS) method for adjusting additional parameters. The ANFIS enables the generation of an interpretable law in the form of a TS model, providing sets of linear parameters (consequent parameters), non-linear parameters (premise parameters), and membership functions (MFs). Given that the vehicle controller has two outputs, it is divided into two multi-input single-output (MISO) subsystems for the application of the ANFIS.

### 2.3. Validate the Learned Controller through Simulation

The closed-loop validation of autonomous vehicles is based on the conformity between the outcomes of the ANFIS controller and those attained by the MPC controller. The precise alignment during the simulation serves as a validation of the proposed approach. However, compared to the MPC controller, the proposed approach has the merit of removing the necessity of online optimization, thereby reducing computational complexity. Stability and performance are assessed in the next step.

### 2.4. Obtain the Takagi–Sugeno Model of the Control Model and Vehicle Model

**Figure 2:**
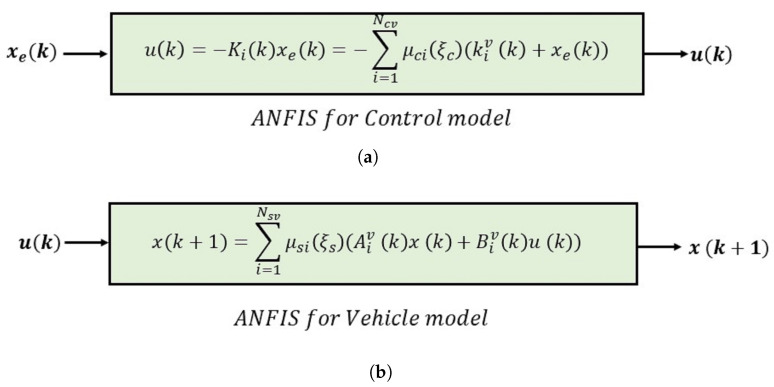
Illustrations of the ANFIS Takagi–Sugeno (TS) representations for (**a**) the control model and (**b**) the vehicle model. A detailed explanation of the parameters is provided in Section 3.

After obtaining, learning, and validating the controller, our objective is to derive the explicit formula for the control law. The procedure is based on performing some inverse steps that the ANFIS internally performs. While the algorithm efficiently computes the consequent and premise parameters, we build two polytopic TS state-space representations for the control model (see Figure 2a) and the vehicle model (see Figure 2b). The vehicle model uses its related ANFIS structure, which is essential for evaluating the stability of the vehicle closed-loop system using the ANFIS controller.

### 2.5. Stability Proof of the Closed-Loop System

The final step involves analyzing the stability of the autonomous vehicle closed-loop system using the ANFIS controller. This is achieved by employing all vertices of both TS representations of the control and vehicle models and applying the Lyapunov stability theorem to the closed-loop system using LMIs [25]. This is presented in detail later in Section 3.7.

## 3. Learning-Based Control Design Description

### 3.1. Considered Autonomous Vehicle

**Figure 3:**
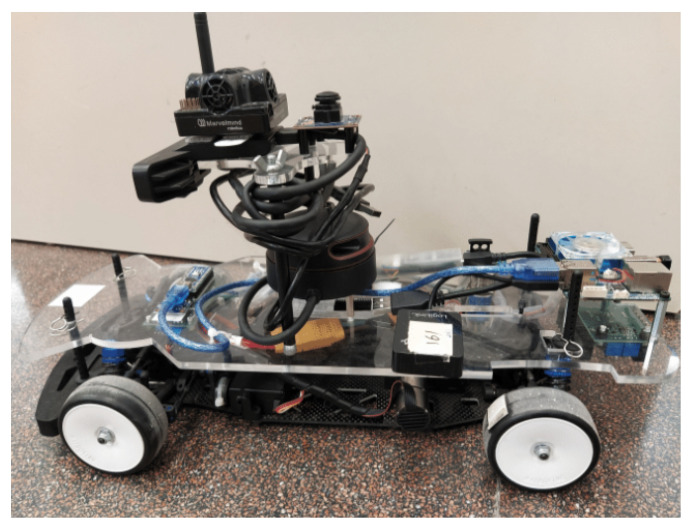
A real picture of the autonomous vehicle used for simulation.

The case study considered is an autonomous race car, which is a developed platform for autonomous driving. This is a rear-wheel drive (RWD) electric remote control (RC) vehicle (see Figure 3) that has been modified to operate autonomously. Mechanically speaking, it has been equipped with some decks to protect the on-board electronics and sensors [47].

**Figure 4:**
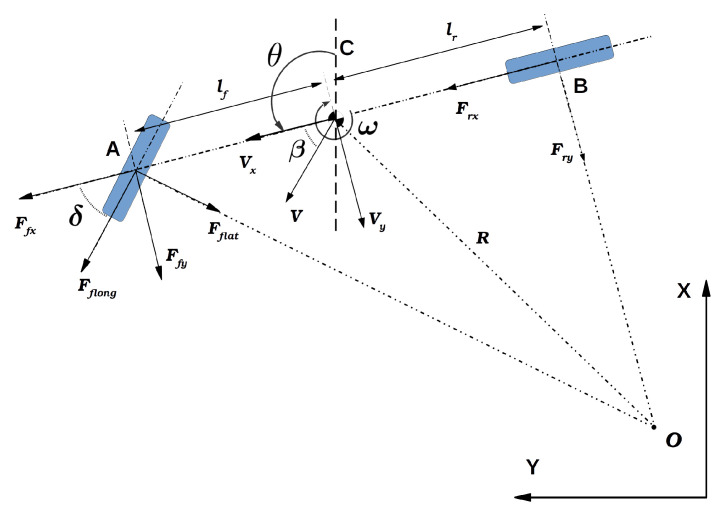
Representation of the bicycle model in 2D space.

The non-linear model used for simulating the considered autonomous vehicle is based on the bicycle model presented in Figure 4 and introduced in [48]:

(1) $$\begin{array}{cc} \; & {\dot{v}}_{x}=a_{r}+\frac{-F_{yf}\sin\delta -\mu g}{m}+\omega v_{y}\\ \; & {\dot{v}}_{y}=\frac{F_{yf}\cos\delta +F_{yr}}{m}-\omega v_{x}\\ \; & \dot{\omega }=\frac{F_{yf}l_{f}\cos\delta -F_{yr}l_{r}}{I}\\ \; & \alpha_{f}=\delta -\tan^{-1}\left(\frac{v_{y}}{v_{x}}-\frac{l_{f}\omega }{v_{x}}\right)\\ \; & \alpha_{r}=-\tan^{-1}\left(\frac{v_{y}}{v_{x}}+\frac{l_{r}\omega }{v_{x}}\right)\\ \; & F_{yf}=d\sin\left(c\tan^{-1}\left(b\alpha_{f}\right)\right)\\ \; & F_{yr}=d\sin\left(c\tan^{-1}\left(b\alpha_{r}\right)\right) \end{array}\;$$

Table 1 lists the system variables. The lateral forces produced in the front and rear tires, denoted $F_{yf}$ f and $F_{yr}$, are determined using the simplified “Magic Formula” model to simulate lateral tire forces. The parameters *b*, *c*, and *d* in this model shape the force curve and are obtained through an identification procedure. The front and rear sliding angles are represented as $\alpha_{f}$ and $\alpha_{r}$, while *m* and *I* represent the mass and inertia of the vehicle. Furthermore, $l_{f}$ and $l_{r}$ are the distances from the vehicle center of mass to the front and rear wheel axes, respectively. The static friction coefficient and gravity constant are denoted as μ and *g*. Table 2 lists the specific values for all dynamic vehicle parameters.

**Table 1:** List of symbols.

Symbol	Description
$v_{x}$	Longitudinal velocity of the vehicle in the center of gravity (CoG) frame (C) in $\left(\frac{m}{s}\right)$; see Figure 4.
$v_{y}$	Lateral velocity of the vehicle in the (CoG) frame (C) in $\left(\frac{m}{s}\right)$.
ω	Angular velocity of the vehicle in the (CoG) frame (C) in $\left(\frac{rad}{s}\right)$.
*X*	Global position of the vehicle in the *x*-axis frame (O) in (m).
*Y*	Global position of the vehicle in the *y*-axis frame (O) in (m).
θ	Orientation of the vehicle with respect to the *x*-axis of the frame (O) in (rad).
*a*	Longitudinal acceleration vector on the rear wheels in $\left(\frac{m}{s^{2}}\right)$.
δ	Steering angle on the front wheels in (rad).

**Table 2:** Model parameters.

Parameter	Value	Parameter	Value
$l_{f}$	0.1377 m	$C_{f}$	45
$l_{r}$	0.1203 m	$C_{r}$	45
*m*	2.424 kg	*I*	0.02 kg $m^{2}$
*b*	6.0	*c*	1.6
*d*	7.76	μ	0.006

### 3.2. Generate the Input and Output Data

The autonomous vehicle equipped with an MPC controller, as described in [47], is used as the data generator. The MPC problem is formulated as a quadratic optimization problem that is solved at each time *k* to determine control actions, given the values of $x_{s}\left(k\right)$ and u(k−1):

(2) $$\begin{array}{cc} \; & \underset{\Delta U(k)}{min}J(k)=\sum_{i=0}^{H_{P}-1}((r\left(k+i\right)-x_{s}\left(k+i\right){)}^{T}Q(r\left(k+i\right)-x_{s}\left(k+i\right))\\ & \;\;\;\cdots +\Delta u{\left(k+i\right)}^{T}R\Delta u\left(k+i\right))+x_{s}{\left(k+H_{p}\right)}^{T}Px_{s}\left(k+H_{p}\right)\\ \; & s.t.:\;\;x_{s}\left(k+i+1\right)=\sum_{j=1}^{N_{\nu }}\mu_{sN_{j}}\left(\zeta_{s}\left(k\right)\right)(A_{j}\hat{x_{s}}\left(k+i\right)+B_{j}u\left(k+i\right)+C_{j})\\ \; & \;\;\;u(k+i)=u(k+i-1)+\Delta u(k+i)\\ \; & \;\;\;\Delta U(k)\in \Delta \prod \\ \; & \;\;\;\Delta \prod =\left\{\Delta u\left(k\right)|A_{\Delta u}\Delta u\left(k\right)=b_{▵u},\Delta u\left(k\right)\geq 0\right\}\\ \; & \;\;\;U(k)\in \prod \\ \; & \;\;\;\prod =\left\{u\left(k\right)|A_{u}u\left(k\right)=b_{u},u\left(k\right)\geq 0\right\}\\ \; & \;\;\;x_{s}\left(k+H_{p}\right)\in \chi \\ \; & \;\;\;y_{e}\in \left[\underline{y_{e}},\bar{y_{e}}\right]\\ \; & \;\;\;x_{s}\left(k\right)=\hat{x_{s}}\left(k\right) \end{array}\;$$

where $\zeta_{s}$:= [$v_{x}$ $v_{y}$ ω δ *a*] is the vector of vehicle scheduling variables, $\hat{x}$ is the estimated state vector, $r={\left[v_{xr}\;0\;\omega_{r}\right]}^{T}$ is the reference vector provided by the trajectory planner, and $H_{p}$ is the prediction horizon. The tuning matrices $Q\in R^{3\times 3}$ and $R\in R^{2\times 2}$ are positive definite in order to obtain a convex cost function. Thus, the values of the variables $x_{c}=\left[v_{xe}\;v_{ye}\;\omega_{e}\right]\;$ and $u={\left[\delta \;a\right]}^{T}$ are recorded as inputs and outputs during the operation of the autonomous vehicle with the MPC controller. These data are employed as training data for the ANFIS in the following step.

### 3.3. Learn the Control Law from Data Using the ANFIS Algorithm 

**Figure 5:**
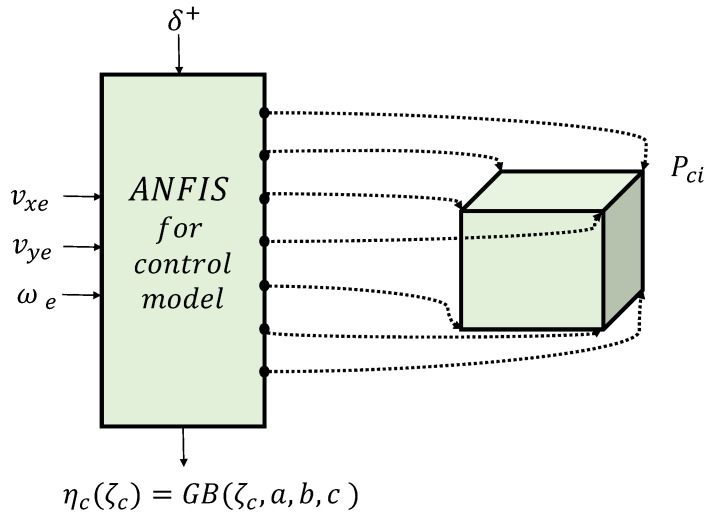
Control model TS representation: TS polytopic learning ANFIS scheme for subsystem δ, using the Gaussian Bell membership function with parameters *a*, *b*, and *c*.

This section outlines the methodology used to obtain the TS representation of the autonomous vehicle control model (see Figure 5). To achieve this, the ANFIS is utilized to learn the structure from the input and output data. In more detail, it learns the MPC controller behavior of the vehicle from the input and output data using the backpropagation technique and a set of membership functions (MFs). A typical membership function is the generalized Gaussian Bell (GB) function. The ANFIS algorithm can only be used for multi-input single-output (MISO) systems. Thus, the control model, which has two outputs, is split into two MISO subsystems to apply the ANFIS. Since our control model is a second-order system, two subsystems are obtained and two learning procedures are carried out. To do this, first, the polynomial representation of each subsystem is formulated as:

(3) $$\begin{array}{cc} \; & P_{ci}=p_{c1i}v_{xe}+p_{c2i}v_{ye}+p_{c3i}\omega_{e}+p_{c4i}\\ \; & \forall i=1,\ldots ,N_{c\upsilon } \end{array}\;$$

where $P_{ci}$ is a linear polynomial representation of the controller of a subsystem at a particular output configuration. $P_{cji}$, $\forall j=1,\ldots ,N_{c\zeta }$ represent the consequent parameters obtained from the ANFIS; $N_{c\zeta }$ is the number of scheduling variables; and $N_{c\upsilon }$ represents the number of polytopic vertices. $v_{xe}$, $v_{ye}$, and $\omega_{e}$ are the trajectory errors that are used as controller inputs. Reorganizing the terms in this equation yields

(4) $$\begin{array}{cc} \; & P_{ci}=\left[p_{c1i}\;p_{c2i}\;p_{c3i}\right]x_{c}+\left[p_{c4i}\right] \end{array}\;$$

where $x_{c}={\left[{v_{x}}_{e}\;\;{v_{y}}_{e}\;\;\;\omega_{e}\right]}^{T}$ is the controller state, and the polynomial structure is transformed into the discrete-time controller representation given by

(5) $$\begin{array}{cc} \; & u_{i}\left(k\right)=-\left(K_{i}\left(k\right)x_{e}\left(k\right)+C_{ci}\left(k\right)\right)\\ \; & \forall i=1,\ldots ,N_{c\upsilon } \end{array}\;$$

where step $u_{i}\left(k\right)$ is the output of subsystem *i*. $K_{i}$ and $C_{ci}$ define the so-called vertex systems, with $u={\left[\delta \;\;a\right]}^{T}$. The generalized Gaussian Bell (GB) membership function, which is defined by three parameters (*a*, *b*, and *c*), is employed as a membership function.

(6) $$\begin{array}{c} \eta_{cm}=\frac{1}{1+{\frac{\zeta_{co}-c_{mo}}{a_{mo}}}^{2b_{mo}}}\\ \forall m=1,\ldots ,N_{cMF},\forall o=1,\ldots ,N_{c\zeta } \end{array}\;$$

where $\zeta_{c}$ represents the ANFIS input vector of the variables and is referred to as the scheduling variables. Moreover, $N_{cMF}$ and $N_{c\zeta }$, respectively, represent the number of controller MFs per scheduling variable and the number of scheduling variables. In this case, if $N_{cMF}$ is two, the normalized weights $\eta_{cNi}$ are computed as follows:

(7) $$\begin{array}{cc} \; & \mu_{ci}\left(\zeta_{c}\right)=\prod_{j=1}^{N_{c\zeta }}\xi_{cij}\left(\eta_{c0},\eta_{c1}\right)\\ \; & \forall i=1,\ldots ,N_{c\upsilon } \end{array}\;$$

where $\xi_{cij}\left(0\right)$ represents any of the weighting functions that depend on each rule *i*. Then, by applying

(8) $$\begin{array}{cc} \; & \mu_{cNi}\left(\zeta_{c}\right)=\frac{\mu_{ci}\left(\zeta_{c}\right)}{\sum_{j=1}^{N_{c\upsilon }}\mu_{cj}\left(\zeta_{c}\right)}\\ \; & \forall i=1,\ldots ,N_{c\upsilon } \end{array}\;$$

the normalized weights are obtained. Each scheduling variable $\zeta_{co}$ is known and varies within a defined interval $\zeta_{co}\in \left[\underline{\zeta_{co}},\bar{\zeta_{co}}\right]\in R$. Finally, the polytopic TS model for each subsystem is represented as:

(9) $$\begin{array}{cc} \; & u_{j}\left(k\right)=-\sum_{i=1}^{N_{c\upsilon }}\mu_{cNji}\left(\zeta_{c}\left(k\right)\right)\left(K_{ji}^{v}\left(k\right)x_{e}\left(k\right)+C_{cji}^{v}\left(k\right)\right)\\ \; & \forall i=1,\ldots ,N_{cG} \end{array}\;$$

where $N_{cG}$ is the number of subsystems. Accordingly, the overall TS system is represented as follows:

(10) $$\begin{array}{cc} \; & u_{j}\left(k\right)=-\sum_{i=1}^{N_{c\upsilon }}\mu_{cNji}\left(\zeta_{c}\left(k\right)\right)\left(\left[\begin{array}{c} K_{1i}^{v}\left(k\right)\\ K_{2i}^{v}\left(k\right) \end{array}\right]x_{e}\left(k\right)+\left[\begin{array}{c} C_{c1i}^{v}\left(k\right)\\ C_{c2i}^{v}\left(k\right) \end{array}\right]\right) \end{array}\;$$

For the sake of clarity, Equation (10) can be expressed as:

(11) $$\begin{array}{cc} \; & u\left(k\right)=-\sum_{i=1}^{N_{c\upsilon }}\mu_{cNi}\left(\zeta_{c}\left(k\right)\right)\left(K_{i}^{v}\left(k\right)x_{e}+C_{ci}^{v}\left(k\right)\right) \end{array}\;$$

and the ANFIS controller gains $K\in R^{2\times 3}$ in Equation (5) are given by

(12) $$\begin{array}{cc} \; & K_{i}\left(k\right)=\sum_{i=1}^{N_{c\upsilon }}\left[\begin{array}{c} \mu_{cN1i}\left(\zeta_{c}\left(k\right)\right)K_{1i}^{v}\left(k\right)\\ \mu_{cN2i}\left(\zeta_{c}\left(k\right)\right)K_{2i}^{v}\left(k\right) \end{array}\right] \end{array}\;$$

### 3.4. Validate the Learned Controller through Simulation

In this step, the closed-loop validation methodology of the ANFIS controller is presented. It is executed through simulation under conditions identical to those under which the MPC controller operates. This means that the ANFIS controller is replaced with the MPC controller as follows:

(13) $$\begin{array}{cc} \; & \delta =evalfis(outFIS\_\delta ,\left[v_{xe}\;v_{ye}\;\omega_{e}\right])\\ \; & a=evalfis(outFIS\_a,\left[v_{xe}\;v_{ye}\;\omega_{e}\right]) \end{array}\;$$

where *evalfis* is the Evaluate Fuzzy Inference System function in the MATLAB fuzzy toolbox; outFIS_δ and outFIS_a are the fuzzy inference systems (FISs) to be evaluated, specified as TS fuzzy inference systems, as described in Section 3.3, and $x_{e}={\left[v_{xe}\;\;v_{ye}\;\omega_{e}\right]}^{T}$ and $u={\left[\delta \;\;a\right]}^{T}$ are, respectively, the inputs and outputs of the ANFIS controller. The outcomes obtained with the ANFIS controller are expected to closely follow those achieved with the MPC controller.

### 3.5. TS Representation for Control Model

Once the algorithm discussed in Section 3.3 has computed the consequent and premise parameters for each of the MISO subsystems, we construct the polytopic TS representation for the control model (for each one of the MISO subsystems). This results in an explicit formula for the control law (see Equation (12)). This TS model has to be validated through the conformity of the parameters obtained with those achieved by the ANFIS. Using the same approach, the TS representation of vehicle states (vehicle model) is achieved in the next step.

### 3.6. TS Representation for Vehicle Model

**Figure 6:**
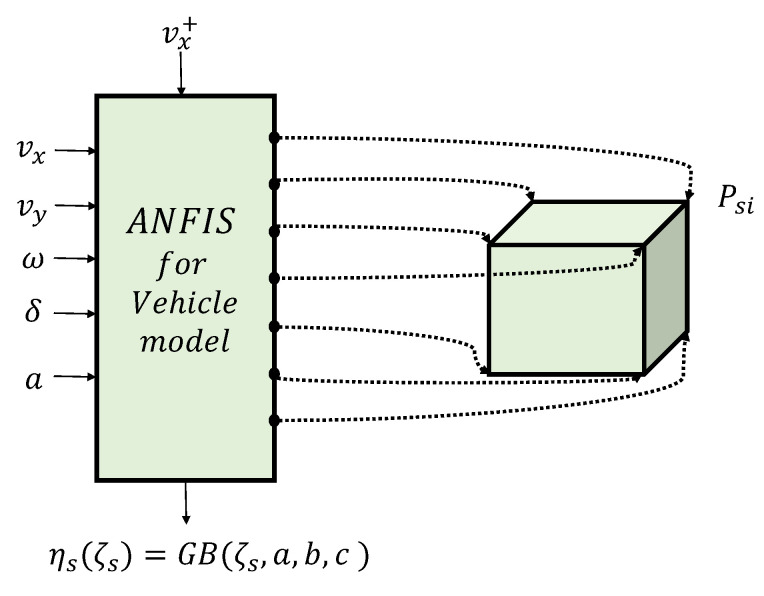
Vehicle model TS representation: TS polytopic learning ANFIS scheme for subsystem $v_{x}$, using the Gaussian Bell membership function with parameters *a*, *b*, and *c* [47].

Obtaining the state space of the vehicle (*vehicle model*) is required for the stability assessment of the system. This can be accomplished through two approaches: the TS approach [47,49] and the linear parameter-varying (LPV) approach [50]. In this work, the vehicle model is represented by the TS model, as shown in Figure 6.

(14) $$\begin{array}{cc} \; & P_{si}=p_{s1i}v_{x}+p_{s2i}v_{y}+p_{s3i}\omega +p_{s4i}\delta +p_{s5i}a+p_{s6i}\\ \; & \forall i=1,\ldots ,N_{s\upsilon } \end{array}\;$$

where the linear polynomial $P_{si}$ represents the output configuration of the state of the vehicle for a particular subsystem. $P_{sji}$, $j=1,\ldots ,N_{s\zeta }$ represent the consequent parameters obtained from the ANFIS; $N_{s\zeta }$ is the number of scheduling variables; $N_{s\upsilon }$ represents the number of polytopic vertices; and $v_{x}$, $v_{y}$, and ω are the system states, which, respectively, represent the longitudinal speed, lateral speed, and angular velocity of the vehicle at each time step. Equation (14) can be rewritten as follows:

(15) $$\begin{array}{cc} \; & P_{si}=\left[p_{s1i}\;p_{s2i}\;p_{s3i}\right]x_{s}+\left[p_{s4i}\;p_{s5i}\right]u+\left[p_{s6i}\right] \end{array}\;$$

where $x_{s}={\left[v_{x}\;v_{y}\;\omega \right]}^{T}$ represents the vehicle state with discrete-time representation

(16) $$\begin{array}{cc} \; & x_{i}\left(k+1\right)=A_{i}x\left(k\right)+B_{i}u\left(k\right)+C_{si}\\ \; & \forall i=1,\ldots ,N_{s\upsilon } \end{array}\;$$

where $A_{i}$, $B_{i}$, and $C_{si}$ are vertex systems, and $u={\left[\delta \;a\right]}^{T}$. The generalized Gaussian Bell (GB) membership function is defined by three parameters (*a*, *b*, and *c*), as follows

(17) $$\begin{array}{cc} \; & \eta_{sm}=\frac{1}{1+{\frac{\zeta_{so}-c_{mo}}{a_{mo}}}^{2b_{mo}}}\\ \; & \forall m=1,\ldots ,N_{sMF},\forall o=1,\ldots ,N_{s\zeta } \end{array}\;$$

where $\zeta_{s}$ represents the ANFIS input vector of the variables or scheduling variables and $N_{sMF}$ and $N_{s\zeta }$, respectively, represent the number of membership functions per scheduling variable and the number of scheduling variables. In this case, the normalized weights $\eta_{sNi}$ are computed as follows:

(18) $$\begin{array}{cc} \; & \mu_{si}\left(\zeta_{s}\right)=\prod_{j=1}^{N_{s\zeta }}\xi_{sij}\left(\eta_{s0},\eta_{s1}\right)\\ \; & \forall i=1,\ldots ,N_{s\upsilon } \end{array}\;$$

where $\xi_{sij}$ stands for any of the weighting functions that depend on each rule *i*

(19) $$\begin{array}{cc} \; & \mu_{sNi}\left(\zeta_{s}\right)=\frac{\mu_{si}\left(\zeta_{s}\right)}{\sum_{j=1}^{N_{s\upsilon }}\mu_{sj}\left(\zeta_{s}\right)}\\ \; & \forall i=1,\ldots ,N_{s\upsilon } \end{array}\;$$

Each scheduling variable $\zeta_{so}$ is known and varies within a defined interval $\zeta_{so}\in \left[\underline{\zeta_{so}},\bar{\zeta_{so}}\right]\in R$. Finally, the polytopic TS model for each subsystem is

(20) $$\begin{array}{cc} \; & x_{sj}\left(k+1\right)=-\sum_{i=1}^{N_{s\upsilon }}\mu_{sNji}\left(\zeta_{s}\left(k\right)\right)\left(A_{ji}^{v}\left(k\right)x\left(k\right)+B_{ji}^{v}\left(k\right)u\left(k\right)+C_{sji}^{v}\left(k\right)\right)\\ \; & \forall i=1,\ldots ,N_{sG} \end{array}\;$$

where $N_{sG}$ is the number of subsystems. The overall TS system is represented as:

(21) $$\begin{array}{cc} \; & x_{sj}\left(k+1\right)=\sum_{i=1}^{N_{s\upsilon }}\mu_{sNji}\left(\zeta_{s}\left(k\right)\right)\left(\left[\begin{array}{c} A_{1i}^{v}\left(k\right)\\ A_{2i}^{v}\left(k\right)\\ A_{3i}^{v}\left(k\right) \end{array}\right]x\left(k\right)+\left[\begin{array}{c} B_{1i}^{v}\left(k\right)\\ B_{2i}^{v}\left(k\right)\\ B_{3i}^{v}\left(k\right) \end{array}\right]u\left(k\right)+\left[\begin{array}{c} C_{1i}^{v}\left(k\right)\\ C_{2i}^{v}\left(k\right)\\ C_{3i}^{v}\left(k\right) \end{array}\right]\right) \end{array}\;$$

For clarity of presentation, Equation (21) can be expressed as

(22) $$\begin{array}{cc} \; & x_{s}\left(k+1\right)=\sum_{i=1}^{N_{s\upsilon }}\mu_{sNi}\left(\zeta_{s}\left(k\right)\right)\left(A_{i}^{v}\left(k\right)x\left(k\right)+B_{i}^{v}\left(k\right)u\left(k\right)+C_{si}^{v}\left(k\right)\right) \end{array}\;$$

and the matrices $A\in R^{3\times 3}$ and $B\in R^{3\times 2}$ are as follows:

(23) $$\begin{array}{cc} \; & A_{i}\left(k\right)=\sum_{i=1}^{N_{s\upsilon }}\left[\begin{array}{c} \mu_{sN1i}\left(\zeta_{s}\left(k\right)\right)A_{1i}^{v}\left(k\right)\\ \mu_{sN2i}\left(\zeta_{s}\left(k\right)\right)A_{2i}^{v}\left(k\right)\\ \mu_{sN3i}\left(\zeta_{s}\left(k\right)\right)A_{3i}^{v}\left(k\right) \end{array}\right] \end{array}\;$$

(24) $$\begin{array}{cc} \; & B_{i}\left(k\right)=\sum_{i=1}^{N_{s\upsilon }}\left[\begin{array}{c} \mu_{sN1i}\left(\zeta_{s}\left(k\right)\right)B_{1i}^{v}\left(k\right)\\ \mu_{sN2i}\left(\zeta_{s}\left(k\right)\right)B_{2i}^{v}\left(k\right)\\ \mu_{sN3i}\left(\zeta_{s}\left(k\right)\right)B_{3i}^{v}\left(k\right) \end{array}\right] \end{array}\;$$

### 3.7. Stability Assessment Using LMIs

Finally, the stability condition of the closed-loop system using the ANFIS controller is presented and proved.

**Proposition 1.**  *Consider the following TS closed-loop system x(k+1)=(A(k)−B(k)K(k))x(k) in discrete time, where the controller K is the ANFIS controller in TS form *(12)*, and the vehicle model is also expressed in TS form (see Equations *(23)* and *(24)*). Then, according to the Lyapunov stability theorem, the previous TS closed-loop system will be stable if there exists a matrix P>0, $P=P^{T}\in R^{n\times n}$, satisfying*

(25) $$\left[\begin{array}{cc} -P & {\left(A_{i}\left(k\right)-B_{i}\left(k\right)K_{j}\left(k\right)\right)}^{T}\\ A_{i}\left(k\right)-B_{i}\left(k\right)K_{j}\left(k\right) & -P^{-1} \end{array}\right]<0\;\forall i=1,\ldots ,N_{s\upsilon },\forall j=1,\ldots ,N_{c\upsilon }$$

**Proof.**  The stability of the autonomous vehicle using the ANFIS controller is assessed through the Lyapunov stability theorem and LMIs by introducing V(x) as a Lyapunov function [25]:

(26) $$\begin{array}{cc} \; & V\left(x\right)=x^{T}Px\\ \; & \Delta V\;=V\left(x(k+1)\right)-V\left(x(k)\right)\\ \; & \;=x{\left(k\right)}^{T}({\left(A_{i}\left(k\right)-B_{i}\left(k\right)K_{j}\left(k\right)\right)}^{T}P\left(A_{i}\left(k\right)-B_{i}\left(k\right)K_{j}\left(k\right)\right))x\left(k\right)-x{\left(k\right)}^{T}Px\left(k\right)\\ \; & \;=x{\left(k\right)}^{T}({\left(A_{i}\left(k\right)-B_{i}\left(k\right)K_{j}\left(k\right)\right)}^{T}PP^{-1}P\left(A_{i}\left(k\right)-B_{i}\left(k\right)K_{j}\left(k\right)\right)-P)x\left(k\right)<0 \end{array}\;$$

Applying the Schur complement to the previous expression yields

(27) $$\begin{array}{cc} \; & \Delta V=\sum_{i=1}^{N_{s\upsilon }}\mu_{si}\left(\zeta_{s}\right)\sum_{j=1}^{N_{c\upsilon }}\mu_{ci}\left(\zeta_{c}\right)\left[\begin{array}{cc} -P & {\left(A_{i}\left(k\right)-B_{i}\left(k\right)K_{j}\left(k\right)\right)}^{T}\\ A_{i}\left(k\right)-B_{i}\left(k\right)K_{j}\left(k\right) & -P^{-1} \end{array}\right]<0 \end{array}$$

where $N_{c\upsilon }$ and $N_{s\upsilon }$ are, respectively, the number of polytopic vertices employed in the ANFIS structure applied to the controller and vehicle TS models. The membership functions $\mu_{si}\left(\zeta_{s}\right)$ and $\mu_{ci}\left(\zeta_{c}\right)$ are positive between zero and one. In order to guarantee negativity for ΔV, the second term needs to be negative. This leads to the LMI condition (25). □

## 4. Results

### 4.1. Data Generation

**Figure 7:**
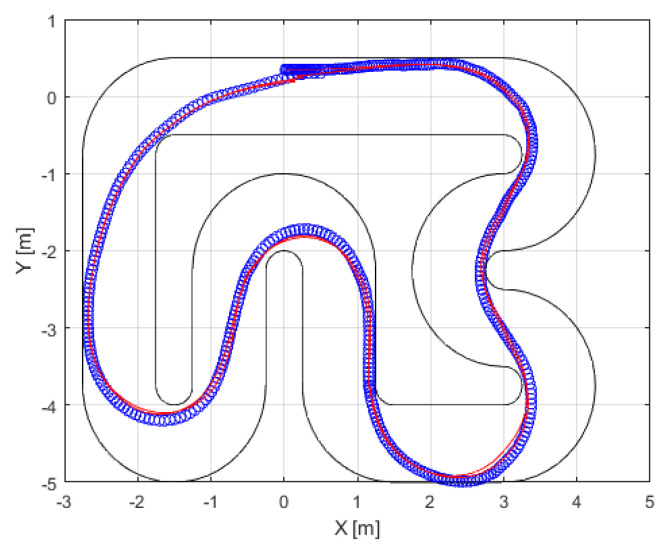
Closed-loopMPC controller simulations, with the positions of the autonomous vehicle system (blue line), trajectory (red line), and track boundaries (black line).

The proposed approach is versatile and can be applied to any type of controller. In this study, we employ an autonomous vehicle system, with the MPC controller (detailed in Section 3.2) as a data generator. The autonomous vehicle (introduced in Section 3.1) was tested on the Verschueren track. Figure 7 provides a visual representation of its operation in the *x*- and *y*-axes. Throughout its operation, both the controller input (trajectory error variables: $x_{c}={\left[v_{xe}\;v_{ye}\;\omega_{e}\right]}^{T}$) and controller output (control action: $u={\left[\delta \;a\right]}^{T}$) were systematically recorded. The diagonal terms for tuning the matrices and input constraints are as follows:

(28) $$\begin{array}{cc} \; & Q=0.65[0.4\;10^{-6}\;0.6]\;R=0.35[0.7\;0.3]\;H_{p}=6\\ \; & A_{u}=\left[\begin{array}{cc} 1 & 0\\ -1 & 0\\ 0 & 1\\ 0 & -1 \end{array}\right]\;A_{\Delta u}=\left[\begin{array}{cc} 1 & 0\\ -1 & 0\\ 0 & 1\\ 0 & -1 \end{array}\right]\;b_{u}=\left[\begin{array}{c} 0.249\\ 0.249\\ 4\\ 1 \end{array}\right]\;b_{\Delta u}=\left[\begin{array}{c} 0.05\\ 0.05\\ 0.5\\ 0.5 \end{array}\right] \end{array}\;$$

### 4.2. Learning the Control Law

**Figure 8:**
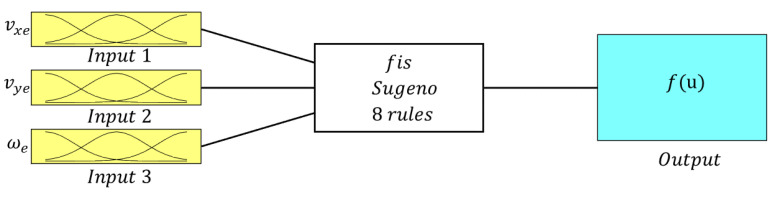
Representation of the ANFIS structure for the controller.

**Figure 9:**
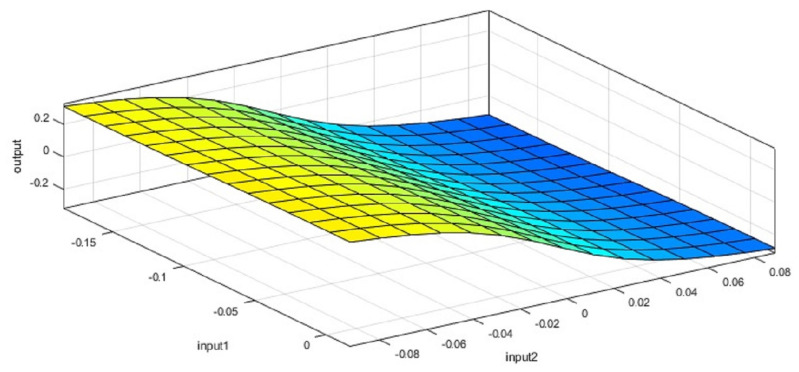
Representation of the fuzzy rules surface for the steering wheel angle (δ). This is one output of the ANFIS controller.The degree of membership is shown in a color contour from blue (lowest) to green (highest).

**Figure 10:**
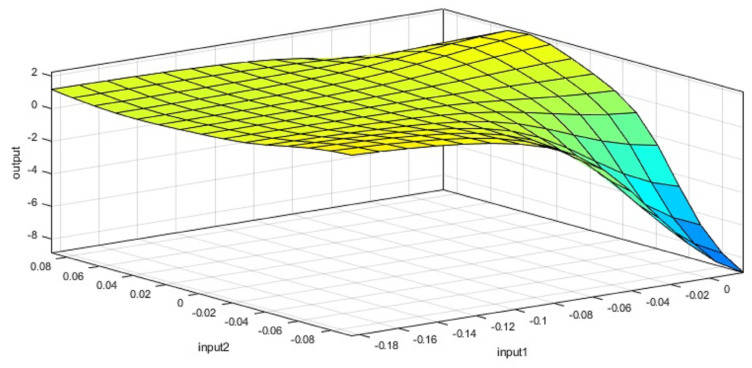
Representation of the fuzzy rules surface for the ANFIS controller acceleration (*a*). This is one output of the ANFIS controller. The degree of membership is shown in a color contour from blue (lowest) to green (highest).

By using the Neuro-Fuzzy Designer app in MATLAB R2021a, a data split of 20% for testing and 80% for training, the hybrid optimization method, and 100 epochs in the training phase, we obtained the ANFIS structure shown in Figure 8 separately for δ and *a*. The resulting neuro-fuzzy rules are illustrated in Figure 9 and Figure 10, corresponding to the fuzzy rules for δ and *a*, respectively. The specifications of the applied ANFIS are also detailed in Table 3.

### 4.3. Validation of the Learned ANFIS Controller

Following the methodology outlined in Section 3.4, the ANFIS controller was integrated into the autonomous vehicle system, and the closed-loop system was simulated. The autonomous vehicle operated under the same conditions as those previously established for the MPC controller.

This simulation involved implementing a control algorithm for a vehicle racing scenario aimed at finding a trajectory within the circuit. It considered a pre-defined trajectory plan along with the constraints of the circuit (Verschueren 2016 map), the ANFIS controller models obtained in Section 4.2, and other vehicle parameters mentioned in Section 3.1. The trajectory points defined the racing track boundaries and reference states, including the velocity, curvature, position, and orientation of the vehicle. The simulation loop iterated over time steps, where at each step, it calculated the errors between the current states and reference states. The simulation used the ANFIS controller (evalfis) to determine control actions based on these errors.

**Figure 11:**
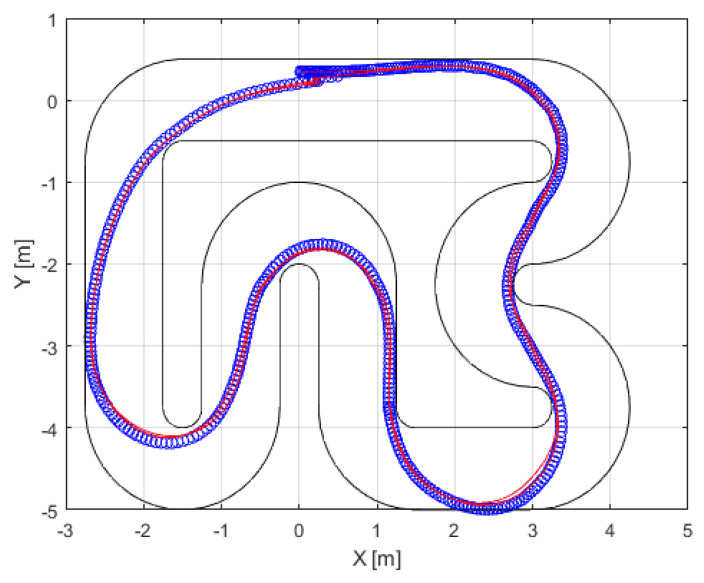
Closed-loop ANFIS controller simulations, with the positions of the autonomous vehicle system (blue line), trajectory (red line), and track boundaries (black line). This operation is known as ANFIS controller design validation.

**Figure 12:**
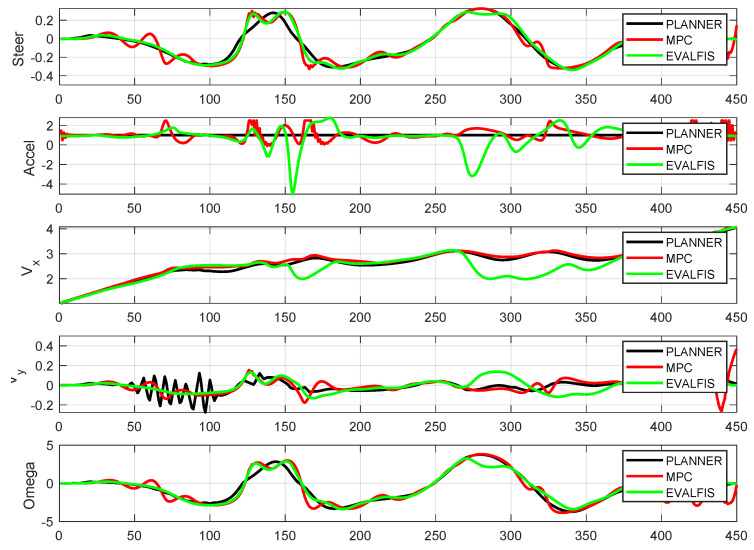
Parameters of the autonomous vehicle system navigating the Verschueren map, using an ANFIS controller (*evalfis*) and an MPC controller. The planner parameters are also highlighted.

**Figure 13:**
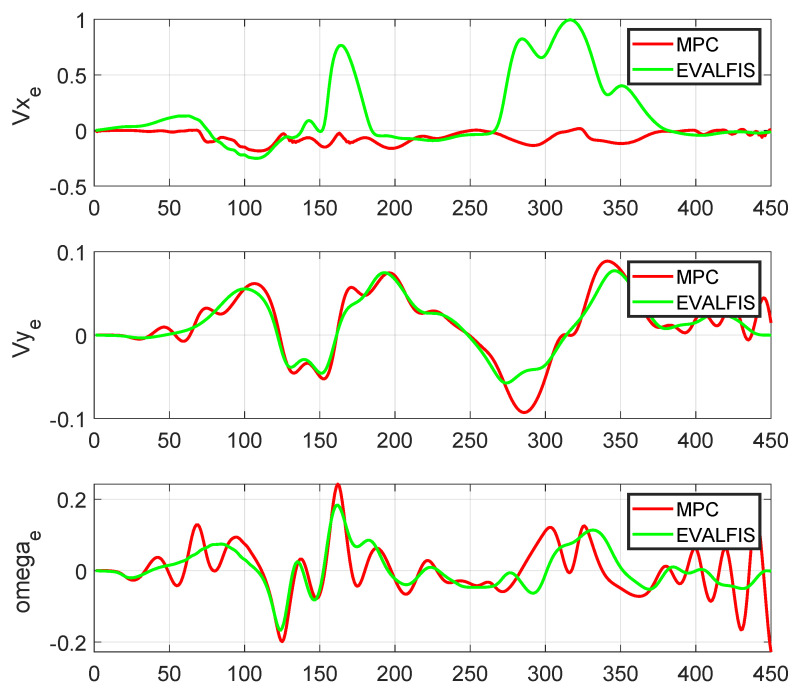
Visualization of the state errors (relative to the planner) for both the ANFIS controller (*evalfis*) and MPC controller.

Figure 11 provides a visual representation of the trajectory followed in the *x*- and *y*-axes. To gain detailed insight into the newly designed controller, we conducted a comparative analysis of the other variables of the autonomous vehicle when using the MPC and ANFIS controllers. Figure 12 depicts a side-by-side comparison of the states and control actions between the reference values provided by the planner and those obtained using these two controllers. Furthermore, Figure 13 shows the state errors (relative to the planner) of both controllers. These errors are quantified as the Mean Square Error (MSE) and are shown in Table 4.

**Figure 14:**
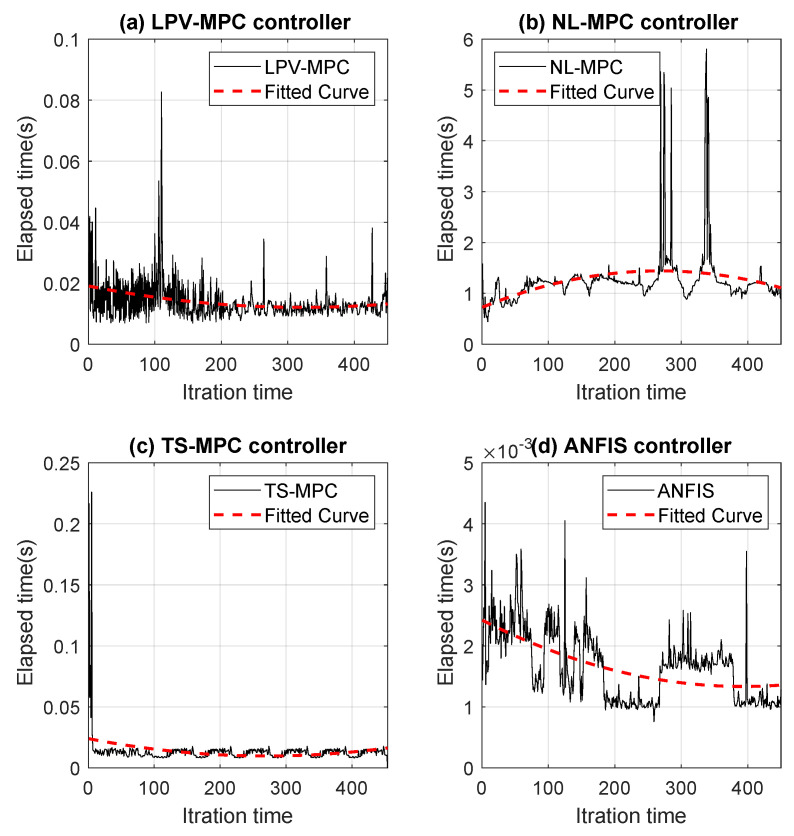
Computational times required for the simulation (one-cycle iteration of the autonomous vehicle on the Verschueren 2016 map) with different controllers: (**a**) LPV-MPC [29], (**b**) NL-MPC [24], (**c**) TS-MPC [45], (**d**) ANFIS.

The trajectory-following performance of the autonomous vehicle was almost the same when using both the ANFIS controller and the MPC controller. Moreover, trajectory errors in two states ($v_{y}$ and ω) were smaller when the system used the ANFIS controller. Differences in the evolution of one state ($v_{x}$) did not affect trajectory tracking. Furthermore, the primary advantage is the notable reduction in computational time. Figure 14 shows the time elapsed for a one-cycle iteration of the autonomous vehicle on the Verschueren 2016 map under the same conditions but with different controllers:

(*a*) MPC controller using LPV to identify the vehicle model (LPV-MPC) [29].
(*b*) MPC controller using the non-linear technique (NL-MPC) [24].
(*c*) MPC controller using TS to identify the vehicle model (TS-MPC) [45].
(*d*) ANFIS controller using TS to identify the control and vehicle models.

**Figure 15:**
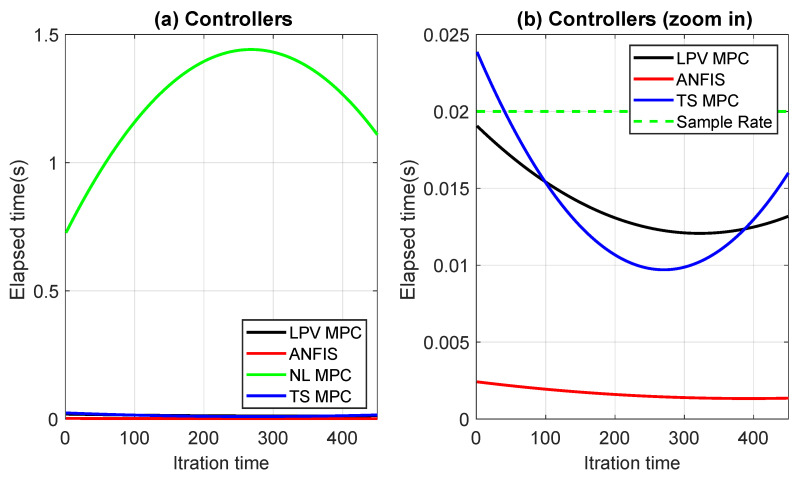
(**a**) Comparison of computational times for all controllers; (**b**) Comparison of elapsed times for controllers operating at a sampling rate of around 20 ms.

Figure 15 depicts a comparison of the controllers, where subfigure (a) shows that the NL-MPC operated at a higher sampling rate of 20 ms, while the other controllers operated below that rate. However, subfigure (b) shows that the ANFIS controller was approximately 10 times faster than the LPV-MPC and TS-MPC controllers.

### 4.4. Validated Takagi–Sugeno (TS) Representation for Both Control and Vehicle Models

**Figure 16:**
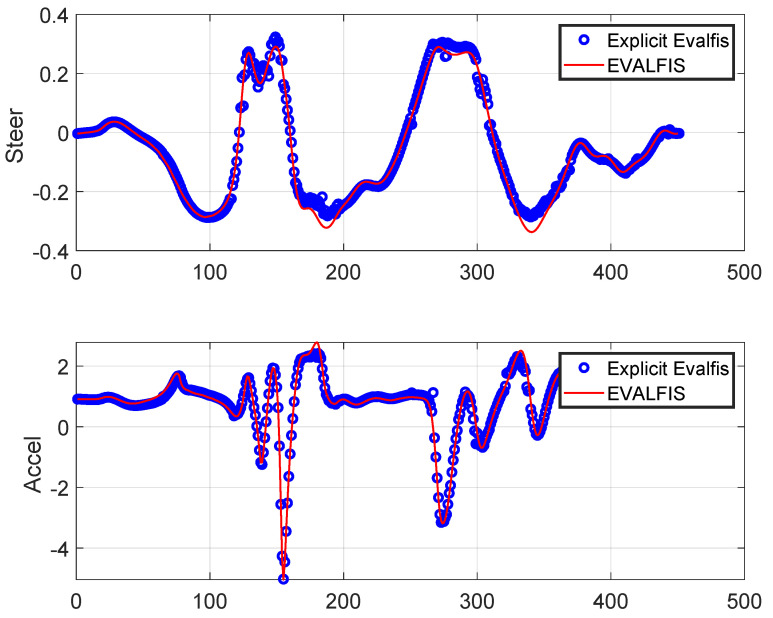
TS representation and validation for the control model: The control actions calculated by the TS model (explicit *evalfis*) closely match those calculated by the ANFIS (*evalfis*).

**Figure 17:**
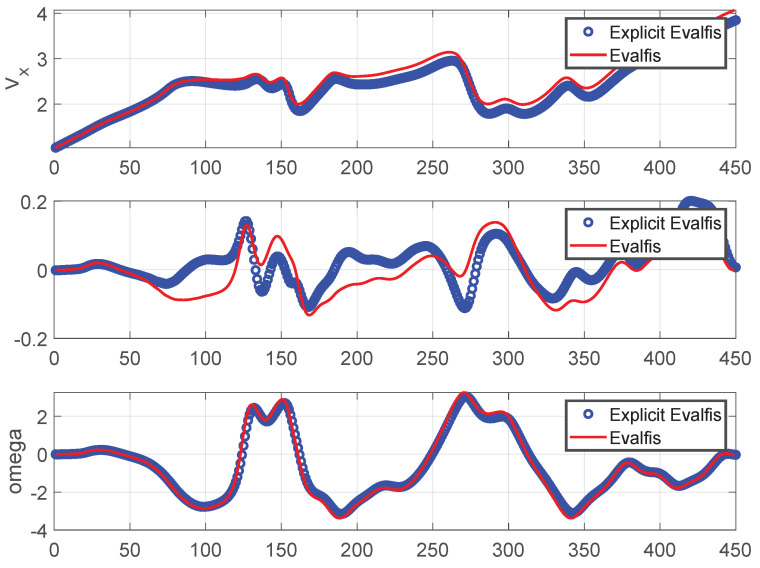
TS representation and validation for the vehicle model: The states calculated by the TS model (explicit *evalfis*) closely match those calculated by the ANFIS (*evalfis*).

The ANFIS output was calculated using the *evalfis* function in Matlab R2021a (from MathWorks, Massachusetts, United States)and the TS explicit representation of the ANFIS control law was also obtained for stability analysis. As a controller, the ANFIS involved three inputs, each associated with two membership functions. This resulted in eight matrices for $K\in R^{2\times 3}$, representing the control model. In addition, the ANFIS was used to obtain the vehicle model, which, in this case, involved three inputs, each associated with two membership functions, resulting in eight matrices of $A\in R^{3\times 3}$ and two matrices of $B\in R^{3\times 2}$. The TS representations for both ANFIS models (controller and vehicle) were obtained using Equations (12), (23), and (24). These TS representations must be verified through the conformity of the TS representation output (explicit *evalfis*) and ANFIS output (*evalfis*). However, there is no guarantee that the TS representation precisely follows the ANFIS output. This validation for the TS representation for the control model is depicted in Figure 16, and for the vehicle model, in Figure 17. The overlap between *evalfis* and the TS explicit representation allowed us to confirm that the TS model corrected both the controller and vehicle.

### 4.5. Stability Assessment

The stability assessment relied on Lyapunov theory, as outlined in Section 3.7. This process utilized YALMIP with SeDuMi 1.3.4, involving 275 LMIs. The assessment concluded the stability of the autonomous vehicle system using the ANFIS controller by establishing a positive-definite matrix *P*, as follows:

$$P=\left[\begin{array}{ccc} 1 & -1.19\times 10^{-12} & 1.15\times 10^{-13}\\ -1.19\times 10^{-12} & 1 & -5.64\times 10^{-12}\\ 1.15\times 10^{-13} & -5.64\times 10^{-12} & 1 \end{array}\right]$$

## 5. Conclusions

In this article, a learning-based approach has been presented to design a controller for an autonomous vehicle under realistic conditions in real time. The ANFIS, as a learning method, is applied to learn a control law using training data obtained from a pre-existing controller. The control law learned from the data is formulated as a Takagi–Sugeno (TS) representation. At the same time, the vehicle model is also learned from the data using the ANFIS. This allows for the proof of closed-loop stability using Lyapunov theory and LMIs for the autonomous vehicle system when the ANFIS controller is used. The proposed approach is validated through simulation using racing-based references provided by an external planner. The ANFIS controller enables the vehicle to perform in racing mode. In comparison to an autonomous vehicle controlled using the MPC controller under the same conditions, the ANFIS controller exhibits reduced errors in two vehicle states ($V_{y}$ and ω) while performing satisfactory trajectory tracking with the added advantage of lower computational time. This strategy has been proposed as an approach to address both autonomous driving control problems and to serve as a parallel controller, enhancing system reliability in the event of a malfunction in the primary controller. The required data in this approach can be obtained from different methods, such as real-world experiments or through simulation. For future work, this approach can be implemented in a real-world experiment using the scale autonomous car and a full-sized vehicle. The application is straightforward since only data from the real vehicle are required. In addition, the proposed method can be applied to the platooning control of multiple vehicles. Some interesting fields of application include the co-design of a bandwidth-aware communication scheduler and cruise controller for multiple high-speed trains, as well as the secure and collision-free multi-platoon control of automated vehicles under data falsification attacks. It is possible to apply the approach using other machine learning methods, such as reinforcement learning [51]. 

## Figures and Tables

**Table 3:** Specifications of the ANFIS architecture.

Name	Type
Generate FIS type	Sugeno
The initial FIS model	Grid partition
Decision method for fuzzy logic operation AND (minimum)	Product
Decision method for fuzzy logic operation OR (maximum)	Probabilistic
Output defuzzification method	Weighted average
Number of membership functions for $v_{xe}$	2
Number of membership functions for $v_{ye}$	2
Number of membership functions for $\omega_{e}$	2
Input membership function type	Gaussian Bell
Output membership function type	Constant
Number of rules	8
Train FIS optimization method	Hybrid
Number of epochs	100

**Table 4:** Trajectory state errors (MSE) relative to the planner for both the ANFIS controller (evalfis) and the MPC controller.

MSE	$v_{\mathrm{xe}}$	$v_{\mathrm{ye}}$	$\omega_{e}$
ANFIS	0.2144	0.0280	0.0417
MPC	0.0587	0.0323	0.0518

## Data Availability

To inquire about the availability of the data used in this study, readers may contact the authors via email.
